# Volume Averaging for Urban Canopies

**DOI:** 10.1007/s10546-019-00470-3

**Published:** 2019-08-17

**Authors:** Manuel F. Schmid, Gregory A. Lawrence, Marc B. Parlange, Marco G. Giometto

**Affiliations:** 1 Department of Civil Engineering, University of British Columbia, Vancouver, BC Canada; 2 Department of Civil Engineering, Monash University, Melbourne, VIC Australia; 3 Department of Civil Engineering and Engineering Mechanics, Columbia University, New York, NY USA

**Keywords:** Double-averaging, Spatial averaging, Urban roughness sublayer, Velocity profiles

## Abstract

When canopy flows are horizontally averaged to obtain mean profiles, the averaging operation can be defined either as an intrinsic average, normalized by the variable fluid volume, or as a superficial average, normalized by the total volume including solid canopy elements. Properties of spatial averages have been explored extensively in the context of flow through plant canopies, albeit with the assumption that the solid volume fraction is negligible. Without this simplification, properties relevant for non-linear terms apply to intrinsic averages while properties of gradients apply to superficial averages. To avoid inconsistencies and inaccuracies the impact of a non-negligible solid volume fraction should be considered carefully when interpreting mean profiles, when deriving mathematical relations for averaged quantities, and when introducing modelling assumptions for such terms. On this basis, we review the definitions and properties of the method of volume averaging, as developed in the more general context of flow through porous media, and discuss its application to urban canopy flows. We illustrate the properties of intrinsic and superficial averages and their effect on mean profiles with example data from a simulation of flow over constant-height cubes.

## Introduction

Averaging has long been a pillar of atmospheric boundary-layer research, and with the help of Reynolds averaging (Reynolds 1895) the complexity of turbulent motions is concentrated in a small number of terms and reduced to a statistical description of the phenomenon (Lumley 1970; Tennekes and Lumley 1972). The resulting time- or ensemble-averaged flow field above the roughness wake layer can often be considered locally homogeneous in the horizontal direction and described with one-dimensional profiles (Parlange et al. 1995). Within and just above the surface roughness, however, this simplicity breaks down. Here, the complex, three-dimensional nature of the flow is not only due to turbulent motions, but also due to the three-dimensional geometry of the surface. Accordingly, equations for one-dimensional profiles obtained by excluding horizontal derivatives no longer hold.

In the study of flow above and within vegetation canopies, the three-dimensionality of the flow was originally addressed with an ad hoc modification of the one-dimensional equations to include a drag force at the level of the plant canopy (see Finnigan and Shaw 2008 for historical details). While such a force had the right effect on the momentum budget, it led to counter-intuitive results such as vegetation always acting to locally suppress turbulence (Wilson and Shaw 1977). Furthermore, the relationship between this force and the three-dimensional flow field was not obvious, as the drag force was not acting at the point at which measurements were taken.

These issues were resolved by deriving canopy equations directly from the full, three-dimensional Navier–Stokes equations by defining a spatial-averaging operation, averaging the flow over a thin horizontal plane (Wilson and Shaw 1977; Raupach and Shaw 1982; Finnigan 1985). With this averaging procedure drag terms arose naturally in the form of integrals over the fluid–solid interface, clarifying their relation to the three-dimensional flow field and their role in higher-order equations.

Interest in flows within urban canopies has been growing. While the irregularity of built environments complicates their study, most human activity takes place in cities, and the growing urban population makes the study of urban flows increasingly relevant (Oke et al. 2017). Many studies have contributed to a better understanding of the flow within urban canopies, ranging from field measurements (e.g. Rotach 1993a, b) to simulations of flow over realistic urban surfaces (e.g. Tseng et al. 2006; Bou-Zeid et al. 2009; Kanda et al. 2013; Giometto et al. 2016, 2017). Special attention has also been directed at the formulation of one-dimensional models for the mean wind profile within urban canopies, based on properties of the surface such as the mean building height or more complex statistics (e.g. Macdonald 2000; Coceal and Belcher 2004; Yang et al. 2016).

Flows over urban canopies are similar to flows over vegetation, and indeed to rough-wall boundary-layer flows in general. Roughness elements provide drag over some vertical distance that acts as a sink for momentum transported down into the roughness layer by turbulent motions. Often the precise location of roughness elements is of little interest and only their aggregate effect on vertical profiles is studied. Therefore, methods and insights from the study of different types of rough surfaces can frequently be shared. Accordingly, much of the work on urban flows has been based on earlier research on flow over vegetation.

However, some fundamental differences between rough surfaces remain. Different aspects of the surface roughness, such as the range of length scales, the sharpness of geometric shapes, and the flexibility of roughness elements, affect the aerodynamic properties of the surface and the resulting flow statistics. As a result, insights from one type of surface roughness cannot always be generalized to surfaces with different types of roughness elements. It appears, for example, that the assumption of an exponential wind profile within the canopy, as proposed by Cionco (1965) for plant canopies, is not appropriate for urban canopies (Castro 2017).

When properties of spatial averages are explored, derivations may rely on surface-specific assumptions. Some of the assumptions commonly made for plant canopies apply to urban surfaces as well. Fluid–solid interfaces are often assumed to be time-invariant and the mean flow is assumed to be locally homogeneous in the horizontal direction after averaging over a sufficiently large scale. Other assumptions are problematic. In much of the literature on volume averaging for plant canopies it is assumed that the volume fraction occupied by solid plant elements is negligible (Finnigan and Shaw 2008). While this may be the case for plant canopies, the assumption is rarely stated explicitly and does not apply to most urban canopies. In a survey of eleven sites in seven North American cities, Grimmond and Oke (1999) reported values between 33% and 58% for the planar area fraction occupied by buildings.

As a result there is no longer a single natural definition of the volume average. Instead, the average can be defined either as the intrinsic average, normalized by the fluid volume that varies as a function of height, or as the superficial average normalized by the total volume including solid regions, constant in height. When the solid volume fraction is zero or negligible, those two definitions become equivalent and adhere to the properties and equations that have been discussed extensively in the literature on flow over vegetation. When the solid volume is non-negligible, however, some of the properties hold for the intrinsic average while others hold for the superficial one. Consequently, both averages are somewhat problematic in the sense that they do not exhibit all the expected or desired properties.

Intrinsic averaging produces values that are more representative of typical values inside the fluid. In the literature on plant canopies, the definition of the volume averaging operation usually corresponds to intrinsic averaging (Ayotte et al. 1999; Finnigan 2000). In the urban literature it is common to see explicit definitions of intrinsic averaging (Martilli and Santiago 2007) as well as references to Finnigan (2000) for the details of spatial averaging (Coceal and Belcher 2004; Belcher 2005; Coceal et al. 2006). However, the behaviour of gradients of intrinsic averages is often problematic due to the fact that both the averaged quantity as well as the fluid volume can change in the direction of the derivative. Intrinsically-averaged gradients give rise to additional terms proportional to the gradient of the fluid volume fraction. For this reason, common forms of budget equations such as the mean momentum equation shown in Finnigan (2000) are incomplete when applied to intrinsic averages in urban canopies. Furthermore, vertically-integrated momentum balances can be defined in multiple ways, none of which is straightforward to interpret, and gradient transport models do not behave in the expected way when the fluid volume changes.

Superficial averaging does not result in such problematic gradients and has also been applied to urban canopy flows (Kono et al. 2009; Lien et al. 2005; Castro et al. 2017; Xie and Fuka 2018). However, the fact that superficial averages are not representative of typical values within the fluid complicates the behaviour of non-linear terms. Products of averages are not representative of averages of products and should be corrected for the fluid volume fraction when deriving averaged equations or when introducing non-linear models for averaged quantities. For similar reasons, a spatial decomposition analogous to the Reynolds decomposition should not be based on superficial averages as this results in spatial correlation terms with unexpected properties. Since these issues only appear for products where both factors have a non-zero mean, they do not affect vertical transport terms if the mean vertical velocity is assumed to be zero. They do become relevant when non-linear models are proposed, or when other non-linear terms such as kinetic energy terms are considered.

For both forms of averaging, mean profiles can become discontinuous when there are step changes in the fluid volume fraction. While this is a special limiting case and real surfaces vary gradually, it deserves some attention due to the fact that perfect cubes are often used as an idealized substitute for real urban surfaces.

In the following sections, we argue that the details of volume averaging should be considered in three different contexts relevant to urban canopy flows. First, care should be taken to apply the correct mathematical properties for each form of averaging when deriving one-dimensional equations from their three-dimensional counterparts. Second, models for one-dimensional profiles should carefully consider how averages are modelled in terms of other averages. When models are derived from three-dimensional models this can be achieved through careful application of mathematical properties. When models are proposed directly for mean quantities, the best choice of averaging can be determined empirically or sometimes through careful deliberation. Third, the impact of the spatial-averaging operation should be considered when interpreting mean profiles. Mean values vary not only due to changes within the fluid but also due to changes in the volume of fluid contributing to the mean and in the composition of different flow regions within the fluid volume. At the very least, every application of spatial averaging should clearly define the averaging operation since there are multiple plausible definitions that behave differently.

In Sect. 2, we review the definitions and properties of volume averaging in the more general context of flow through porous media. In the study of flow through porous media it is difficult to ignore the central importance of the solid volume fraction and its spatial variations for averaged quantities. Similar to the study of plant canopies, early equations were formulated directly for average quantities (e.g. Darcy’s law; Darcy 1856), but when the averaging procedure was formalized, both the solid and fluid phases had to be taken into account. As a result, publications on the averaging for porous media flows contain a detailed treatment of solid and fluid volume fractions as well as the different definitions of volume averages they enable (e.g. Whitaker 1973). Further work discussed related issues such as the proper definition of spatial deviations from the average (Gray 1975) and the differentiability of spatial averages (Howes and Whitaker 1985), and Whitaker (1999) presents a detailed discussion of the method of volume averaging. By considering mean profiles of flow within and above urban surfaces as a special case of volume averaging in porous media, we make use of insights gained for porous media flows in the study of urban canopy flows.

In Sect. 3, we then show how these definitions can be applied to obtain averaged profiles of urban canopy flows and present different versions of important budget equations. This methodology has already been applied successfully in the context of flow over gravel-beds (Nikora et al. 2007; Mignot et al. 2008, 2009). Here, we extend this work to focus on the special case of urban canopy flows. While these flows are similar in nature to gravel-bed flows, certain features such as the horizontal surfaces at the top of cuboid roughness elements deserve particular attention. Furthermore, we present and discuss different versions of the integral form of the momentum balance that is often used instead of the force balance in atmospheric boundary-layer research.

In Sect. 4, we discuss in detail how different terms behave in the idealized setting of flow over constant-height cubes in order to illustrate the influence of the solid volume fraction. The step change at the top of the cubes allows us to discuss how both types of average are affected by variations in the fluid volume fraction and to separate these effects from gradual changes in the nature of the flow. The discussion applies both to idealized flows over cubes, where the contributions often take the form of a delta function, and to realistic urban surfaces, where the geometry changes gradually and the contributions to budget equations are gradual as well.

## Volume Averaging in Porous Media

Flow through urban canopies is a special case of flow through porous media. Since volume averaging has been studied extensively for porous media (cf. Whitaker 1999), the method can be applied to urban canopy flows. We first present a summary of the relevant definitions, assumptions, and properties of volume averaging for a porous medium with a fluid phase flowing through a fixed solid phase.

### Definition of Averaging Region

**Fig. 1:**
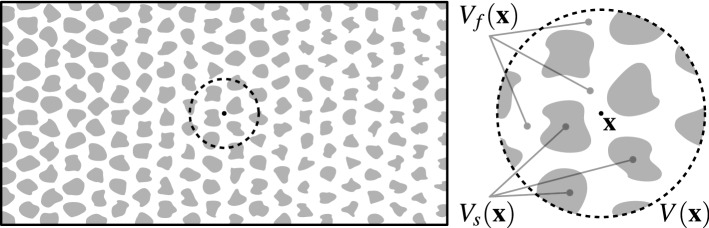
Overview and detail of a porous medium with an averaging region $V$ centred at $\mathbf {x}$ containing both fluid ($V_{f}$, white) and solid ($V_{s}$, grey) regions

The starting point of volume averaging is the definition of an averaging region $V ({\mathbf {x}})$ for each point in space ${\mathbf {x}}$. This region consists of a fluid fraction $V_{f} ({\mathbf {x}})$ and a solid fraction $V_{s} ({\mathbf {x}})$ with $V_{f} ({\mathbf {x}}) \cup V_{s} ({\mathbf {x}}) = V ({\mathbf {x}})$, as illustrated in Fig. 1. We note that the point ${\mathbf {x}}$ can be in either of the two phases. A central feature of volume averaging is that it takes a quantity that is only defined in the fluid region and turns it into a quantity that is defined everywhere. In the process, the complexities of flow features and boundary conditions on scales smaller than the averaging region are removed and only their aggregate effect on the flow is retained.

The same symbols are employed both for the volume as a mathematical domain (e.g. for integration) as well as the size (measure) of this domain; the meaning should be clear from the context. While the size and shape of the averaging region $V$ can in principle be a function of space and time, this is rarely desirable. For the sake of this discussion it is assumed that the size of $V$ is constant since some results make use of this fact. The size and shape of $V_{f}$ and $V_{s}$ are, of course, not constant and depend on the local geometry.

### Definition of Volume Average

We now consider a quantity $\varphi$ defined anywhere in the fluid. Based on the earlier definition of averaging regions, we define two relevant volume averages: the intrinsic average (Eq. 1.2-9 in Whitaker 1999),

1 $$\begin{aligned} {\left\langle \varphi \right\rangle }_{_{ I}}(\mathbf {x})&\equiv \frac{1}{ V_{f} (\mathbf {x})} \int _{\mathbf {y} \in V_{f} (\mathbf {x})} \varphi (\mathbf {y}) \, \mathrm {d}V\text {,} \end{aligned}$$

and the superficial average (Eq. 1.2-7 in Whitaker 1999),

2 $$\begin{aligned} {\left\langle \varphi \right\rangle }_{_{ S}}(\mathbf {x})&\equiv \, \frac{1}{\, V \,} \, \int _{\mathbf {y} \in V_{f} (\mathbf {x})} \varphi (\mathbf {y}) \, \mathrm {d}V\text {.} \end{aligned}$$

Explicitly stating the dependence on ${\mathbf {x}}$ and ${\mathbf {y}}$ allows us to make two observations. First, we note that the average at position ${\mathbf {x}}$, which can be in both fluid or solid regions, depends on values at other positions ${\mathbf {y}}$ within the fluid, away from ${\mathbf {x}}$. While it should be obvious that volume averaging is a non-local procedure, it is worth stating this explicitly as it is important when considering averages of averages, or averages of deviations. Second, we note that the region $V_{f}$ is a function of space too. While the total volume $V$ is chosen to be constant everywhere, the fluid volume $V_{f}$ depends on the configuration of fluid and solid spaces and generally remains a function of ${\mathbf {x}}$.

Both intrinsic and superficial volume averaging have merits. The intrinsic average is often more representative of typical values found in the fluid and can therefore be more comparable to measurements. The superficial average is often more representative of quantities in an integral sense and can be more intuitive in the context of conservation laws. In the case of a constant concentration, for example, the intrinsic average gives the actual concentration found in the fluid whereas the superficial average represents the total mass per unit volume.

Converting between the two definitions is straightforward, with

3 $$\begin{aligned} {\left\langle \varphi \right\rangle }_{_{ S}}(\mathbf {x}) = \varepsilon ({\mathbf {x}}) {\left\langle \varphi \right\rangle }_{_{ I}}(\mathbf {x})\text {,} \end{aligned}$$

where $\varepsilon ({\mathbf {x}}) \equiv V_{f} ({\mathbf {x}}) / V$ is the volume fraction (Eqs. 1.2-6 and 1.2-10 in Whitaker 1999).

Due to this simple relationship, the initial choice of averaging is often secondary. Derivations of averaged budget equations can be based on either of the two definitions as long as this is done consistently. Resulting terms can be expressed in the form most useful for their interpretation. Care has to be taken when one average term is modelled in terms of other averages to ensure that the resulting model scales correctly with different geometries.

### Definition of Decomposition

In the same spirit as the Reynolds decomposition, a quantity can be decomposed into its spatial average and the local departures from this average. This decomposition could in principle be based on either one of the two averages. In practice, however, the sensible choice is the intrinsic average, with

4 $$\begin{aligned} \varphi (\mathbf {x}) \equiv {\left\langle \varphi \right\rangle }_{_{ I}}(\mathbf {x}) + \widetilde{ \varphi } (\mathbf {x}) \end{aligned}$$

defining the spatial deviations $\widetilde{ \varphi }$ (Eq. 1.2-33 in Whitaker 1999).

Even though the spatial average is defined everywhere, the deviations $\widetilde{ \varphi }$ are only defined in the fluid region, just like the quantity $\varphi$ itself. The deviations do however inherit the non-local nature of the average, in the sense that the value of $\widetilde{ \varphi } ({\mathbf {x}})$ is influenced by values of $\varphi$ at locations other than ${\mathbf {x}}$.

The reasons for defining $\widetilde{ \varphi }$ as a departure from the intrinsic rather than from the superficial average have been discussed by Gray (1975). In regions with a non-zero solid volume fraction, the superficial average ${\left\langle \varphi \right\rangle }_{_{ S}}$ is lower than typical values of $\varphi$ in the fluid. Departures from superficial averages are therefore non-zero and spatially correlated even for constant quantities. The magnitudes and correlations of departures are likely to be dominated by the difference between the lower value of the superficial average and the higher values within the fluid, rather than the variation of values within the fluid. Therefore, a decomposition based on the superficial average would complicate rather than facilitate the interpretation and modelling of spatially-averaged equations and is best avoided.

The definition of spatial deviations also sheds light on another issue. Sometimes the spatial average is presented in a slightly different manner, where the quantity $\varphi$ is extended to the solid region with $\varphi ({\mathbf {x}}\in V_{s} )\equiv 0$ and integrated over the whole region $V$ to compute the average. Both a superficial and an intrinsic average can be defined this way, depending on whether the integral is normalized by $V$ or $V_{f}$. This definition may seem more straightforward since both averaged and non-averaged quantities are defined everywhere in space. However, it raises the question of how the spatial decomposition should be defined such that it also applies everywhere. In the fluid region, the considerations discussed above still apply and departures from the superficial average are problematic. In the solid region, the same problems apply to departures from either average, resulting in values that are non-zero even for constant $\varphi$ and producing strong spatial correlations dominated by the difference between fluid and solid regions. For a meaningful interpretation of spatial deviations, $\widetilde{ \varphi }$ would have to be defined as departure from yet another spatial average that takes on the value of the intrinsic average in the fluid region and is zero in the solid region. This complicates the definitions unnecessarily and we therefore avoid extending quantities to solid regions.

### Reynolds Averaging Rules

A convenient property of the ensemble average ${{\overline{\varphi }}} \equiv \frac{1}{N} \sum _{i=1}^N \varphi _i$ often employed in Reynolds averaging is that it acts as a projection, i.e. ${{\overline{ {{\overline{\varphi }}} }}} = {{\overline{\varphi }}}$ as long as the number of realizations $N$ is high enough. This property and related properties, such as ${{\overline{\varphi _1 {{\overline{\varphi _2}}} }}} = {{\overline{\varphi _1}}} \; {{\overline{\varphi _2}}}$ and ${{\overline{ {\varphi ^\prime } }}} =0$ for the random deviations ${\varphi ^\prime } \equiv \varphi - {{\overline{\varphi }}}$, are usually referred to as Reynolds averaging rules. As already discussed by Reynolds (1895), these properties generally do not hold exactly when averaging over a limited range of time or space. Instead, they require additional assumptions on the regularity of the mean field, such as statistical stationarity. Most flows are stationary neither in time nor in space. However, the properties still hold approximately as long as the flow is close to stationary on the time scale of the averaging period or close to homogeneous on the spatial scale of the averaging region.

The restrictions on length scales that permit moving volume averages out of the spatial integral are discussed at length by Whitaker (1999). What is required is a separation of scales between the spatial inhomogeneities that the average is supposed to remove and the scales over which the average varies appreciably. When the averaging region is chosen to lie between these two scales, the intrinsic average acts as a projection. The superficial average produces an additional factor of $\varepsilon$ when applied twice, which follows directly from the fact that ${\left\langle 1 \right\rangle }_{_{ S}}= \varepsilon$, so

5 $$\begin{aligned} {\left\langle {\left\langle \varphi \right\rangle }_{_{ I}} \right\rangle }_{_{ I}} \ne {\left\langle \varphi \right\rangle }_{_{ I}} \quad \text {and} \quad {\left\langle {\left\langle \varphi \right\rangle }_{_{ S}} \right\rangle }_{_{ S}} \ne \varepsilon {\left\langle \varphi \right\rangle }_{_{ S}} \end{aligned}$$

in general, while

6 $$\begin{aligned} {\left\langle {\left\langle \varphi \right\rangle }_{_{ I}} \right\rangle }_{_{ I}} \approx {\left\langle \varphi \right\rangle }_{_{ I}} \quad \text {and} \quad {\left\langle {\left\langle \varphi \right\rangle }_{_{ S}} \right\rangle }_{_{ S}} \approx \varepsilon {\left\langle \varphi \right\rangle }_{_{ S}} \end{aligned}$$

with separation of scales. The corresponding rules for the average of deviations follow from the definitions, so

7 $$\begin{aligned} {\left\langle \widetilde{ \varphi } \right\rangle }_{_{ I}} \ne 0 \quad \text {and} \quad {\left\langle \widetilde{ \varphi } \right\rangle }_{_{ S}} \ne 0 \end{aligned}$$

in general, while

8 $$\begin{aligned} {\left\langle \widetilde{ \varphi } \right\rangle }_{_{ I}} \approx 0 \quad \text {and} \quad {\left\langle \widetilde{ \varphi } \right\rangle }_{_{ S}} \approx 0 \end{aligned}$$

with separation of scales.

Note that the separation of scales can only be valid both for the intrinsic and superficial average if it is also valid for the volume fraction $\varepsilon$. If $\varepsilon$ is not close to uniform on the scale of the averaging region, the same has to be true for at least one of the spatial averages.

### Commutativity of Averaging and Addition/Multiplication

From the definition of volume averaging as an integral, it follows that averages of sums are the same as sums of averages,

9 $$\begin{aligned} {\left\langle \varphi _1 + \varphi _2 \right\rangle }_{_{ I}} = {\left\langle \varphi _1 \right\rangle }_{_{ I}} + {\left\langle \varphi _2 \right\rangle }_{_{ I}} \quad \text {and} \quad {\left\langle \varphi _1 + \varphi _2 \right\rangle }_{_{ S}} = {\left\langle \varphi _1 \right\rangle }_{_{ S}} + {\left\langle \varphi _2 \right\rangle }_{_{ S}}. \end{aligned}$$

Based on the projection properties in the presence of a separation of scales, averages of products can be related to products of averages. As with the familiar Reynolds averaging, this produces an additional correlation term (Eq. 3.2-17 in Whitaker 1999),

10 $$\begin{aligned} {\left\langle \varphi _1 \varphi _2 \right\rangle }_{_{ I}} = {\left\langle \left( {\left\langle \varphi _1 \right\rangle }_{_{ I}} + \widetilde{ \varphi _1 } \right) \left( {\left\langle \varphi _2 \right\rangle }_{_{ I}} + \widetilde{ \varphi _2 } \right) \right\rangle }_{_{ I}} \approx&\,\qquad {\left\langle \varphi _1 \right\rangle }_{_{ I}} {\left\langle \varphi _2 \right\rangle }_{_{ I}} + {\left\langle \widetilde{ \varphi _1 } \widetilde{ \varphi _2 } \right\rangle }_{_{ I}}, \end{aligned}$$

11 $$\begin{aligned} {\left\langle \varphi _1 \varphi _2 \right\rangle }_{_{ S}} = {\left\langle \left( {\left\langle \varphi _1 \right\rangle }_{_{ I}} + \widetilde{ \varphi _1 } \right) \left( {\left\langle \varphi _2 \right\rangle }_{_{ I}} + \widetilde{ \varphi _2 } \right) \right\rangle }_{_{ S}} \approx&\, \varepsilon ^{-1} {\left\langle \varphi _1 \right\rangle }_{_{ S}} {\left\langle \varphi _2 \right\rangle }_{_{ S}} + {\left\langle \widetilde{ \varphi _1 } \widetilde{ \varphi _2 } \right\rangle }_{_{ S}}. \end{aligned}$$

As mentioned above, the spatial deviations are based on the intrinsic average. Otherwise, these new correlation terms could be non-zero even if both quantities are constant everywhere. This would make it difficult to interpret such terms and to construct physically meaningful models for them.

### Commutativity of Averaging and Differentiation

As long as a function and its derivative are continuous and the limits of integration are fixed, integration and differentiation commute (cf. Leibnitz rule). Therefore, volume averages can be moved inside time derivatives as long as the geometry does not change over time,

12 $$\begin{aligned} {\left\langle \frac{\partial \, \varphi }{\partial t} \right\rangle }_{_{ I}} \bigg |_\mathbf {x} = \frac{\partial \, {\left\langle \varphi \right\rangle }_{_{ I}}}{\partial t} \bigg |_\mathbf {x} \quad \text {and} \quad {\left\langle \frac{\partial \, \varphi }{\partial t} \right\rangle }_{_{ S}} \bigg |_\mathbf {x} = \frac{\partial \, {\left\langle \varphi \right\rangle }_{_{ S}}}{\partial t} \bigg |_\mathbf {x}. \end{aligned}$$

This is generally true for urban canopies with solid boundaries, but not always for plant canopies when waving plant motions are to be accounted for. In that case, additional terms would have to be introduced to account for the movement of the boundaries.

For spatial derivatives, the situation is more complicated. Since the domain of integration is a function of space, the averaging does not commute with spatial differentiation. The relation between averages of derivatives and derivatives of averages is given by the spatial averaging theorem (Anderson and Jackson 1967; Slattery 1967; Whitaker 1967; Eq. 1.2-16 in Whitaker 1999),

13 $$\begin{aligned} {\left\langle \frac{\partial \, \varphi }{\partial x_i} \right\rangle }_{_{ S}} \bigg |_\mathbf {x}&= \frac{\partial \, {\left\langle \varphi \right\rangle }_{_{ S}}}{\partial x_i} \bigg |_\mathbf {x} + \frac{1}{ V } \int _{\mathbf {y} \in A_{ f s} (\mathbf {x})} \varphi (\mathbf {y}) \, n_i \, \mathrm {d}A. \end{aligned}$$

The surface integral that appears in (13) can be understood as the average effect of the boundary conditions within the averaging region. $A_{ f s} ({\mathbf {x}})$ is the interface between the fluid and solid regions contained in $V ({\mathbf {x}})$. The surface normal ${\mathbf {n}}$ is oriented such that it points from the fluid region $V_{f}$ into the solid region $V_{s}$; $n_i$ is its component in $x_i$-direction.

The spatial averaging theorem is more easily derived for the superficial average. The corresponding relationship for the intrinsic average can be obtained from ${\left\langle \varphi \right\rangle }_{_{ S}} = \varepsilon {\left\langle \varphi \right\rangle }_{_{ I}}$,

14 $$\begin{aligned} {\left\langle \frac{\partial \, \varphi }{\partial x_i} \right\rangle }_{_{ I}} \bigg |_\mathbf {x}&= \frac{\partial \, {\left\langle \varphi \right\rangle }_{_{ I}}}{\partial x_i} \bigg |_\mathbf {x} + \frac{{\left\langle \varphi \right\rangle }_{_{ I}}}{ \varepsilon } \frac{\partial \, \varepsilon }{\partial x_i} \bigg |_\mathbf {x} + \frac{1}{ V_{f} (\mathbf {x})} \int _{\mathbf {y} \in A_{ f s} (\mathbf {x})} \varphi (\mathbf {y}) \, n_i \, \mathrm {d}A. \end{aligned}$$

The new term is non-zero when the fluid volume considered for the average changes in the direction of the derivative or, equivalently, there is an imbalance in the orientation of fluid–solid interfaces within the averaging region. If the statistical behaviour of $\varphi$ at the interface is the same as within the fluid, the new term and the surface integral cancel. Otherwise, the difference has to be made up by local changes of $\phi$ (contributing to $\langle \partial \varphi /\partial {x_{i}}\rangle _{_{I}}$) or by a non-zero contribution to $\partial {\left\langle \varphi \right\rangle }_{_{ I}}/\partial x_i$.

### Double-Averaging in Time and Space

The temporal variability of flows can be addressed with double-averaging, where a time- or ensemble-average ${{\overline{\varphi }}}$ is performed in addition to the volume averaging (Nikora et al. 2007; Finnigan and Shaw 2008). If ${{\overline{\varphi }}}$ is defined as an integral over a fixed (or infinite) time interval, i.e. ${{\overline{\varphi }}} ({\mathbf {x}}, t) \equiv 1/T \int _{\scriptscriptstyle t-T/2}^{\scriptscriptstyle t+T/2} \varphi ({\mathbf {x}}, t) \, \mathrm {d}t$, or as an ensemble average (cf. Sect. 2.4), and the region $V_{f}$ for spatial averaging is constant in time (or between ensemble runs), the two operations commute and the order in which they are applied does not matter. This is true for averages, for deviations, and for combinations thereof (Pedras and de Lemos 2000),

15 $$\begin{aligned} {\left\langle {{\overline{\varphi }}} \right\rangle }_{_{ I}} = {{\overline{{\left\langle \varphi \right\rangle }_{_{ I}}}}} \, , \quad {\left\langle {\varphi ^\prime } \right\rangle }_{_{ I}} = {{\left\langle \varphi \right\rangle }_{_{ I}}^\prime } \, , \quad \widetilde{ {{\overline{\varphi }}} } = {{\overline{\widetilde{ \varphi } }}} \, , \quad \text {and} \quad \widetilde{ {\varphi ^\prime } } = {\widetilde{ \varphi } ^\prime } . \end{aligned}$$

A complete picture of how the double-averaging affects non-linear terms is obtained when quantities are decomposed into four parts $\varphi = {\left\langle {{\overline{ \varphi }}} \right\rangle }_{_{ I}} + {\left\langle {\varphi ^\prime } \right\rangle }_{_{ I}} + \widetilde{ {{\overline{\varphi }}} } + \widetilde{ {\varphi ^\prime } }$, resulting in four contributions to double-averaged non-linear terms (Pedras and de Lemos 2000),16A double-averaged product can therefore be decomposed into the product of double-averages (term I), the correlation in time of spatial averages (term II), the correlation in space of time averages (term III), and another correlation term that can be understood as the mean correlation in time of spatial deviations or the mean correlation in space of temporal deviations (term IV).

Often the partial decomposition $\varphi = {\left\langle {{\overline{ \varphi }}} \right\rangle }_{_{ I}} + \widetilde{ {{\overline{\varphi }}} } + {\varphi ^\prime }$ is used when the averaging is considered to be performed in time first and $\varphi = {{\overline{{\left\langle \varphi \right\rangle }_{_{ I}}}}} + {{\left\langle \varphi \right\rangle }_{_{ I}}^\prime } + \widetilde{ \varphi }$ when starting with spatial averaging (Nikora et al. 2007). This way, non-linear terms produce a single term for terms II and IV in the first case and for terms III and IV in the latter case. If the averaging region is large enough to include all scales of motion, term II vanishes. In this case it is also possible to consider averaging in space only (Wilson and Shaw 1977), in which case non-linear terms produce a mean product ${\left\langle \varphi _1 \right\rangle }_{_{ I}}{\left\langle \varphi _2 \right\rangle }_{_{ I}}$ equivalent to term I, and a correlation term ${\left\langle \widetilde{ \varphi _1 } \widetilde{ \varphi _2 } \right\rangle }_{_{ I}}$ equivalent to terms III and IV. As all of these decompositions are valid, the choice should be based on whether the interpretation or modelling of terms I–IV is easier if they are separate or combined.

## Volume Averaging in Canopy Layers

The techniques and insights of volume averaging can be employed to derive equations for one-dimensional vertical profiles of flow within and above canopy layers. This way, we avoid ad hoc modifications of boundary-layer equations to account for the effects of the canopy. Instead, new terms arise naturally from the averaging procedure and can be related back to the three-dimensional flow field.

When applied to canopy layers, the goal of the averaging procedure is to reduce a three-dimensional flow field with a three-dimensional surface geometry to a one-dimensional vertical profile that can be used in more practical applications. This is achieved with an appropriate choice of averaging region where the horizontal variability is removed while the vertical structure is retained. In the following, we define such a region and discuss some of the implications before we present the resulting transport equations.

### Definition of Averaging Region

**Fig. 2:**
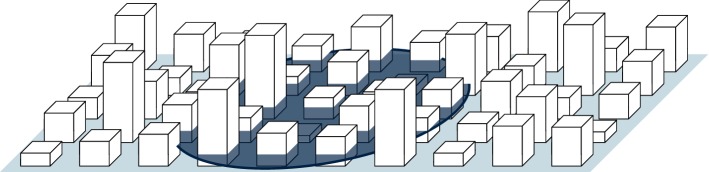
Illustration of the averaging region (shown in dark) used for horizontal averaging in an urban canopy layer

The definition of the averaging region $V$ is driven by mathematical and conceptual considerations. Mathematically, the choice of the averaging region should make it possible to simplify averaged equations and neglect horizontal derivatives as well as mixed terms such as ${\left\langle {\left\langle \varphi _1 \right\rangle }_{_{ I}}\widetilde{ \varphi _2 } \right\rangle }_{_{ I}}$. As discussed in Sects. 2.4 and 2.5, this requires a separation of scales between the scale of the averaging region and the scale over which the averaged quantities vary. Conceptually, the choice of averaging region should enable a sensible interpretation of the resulting profiles, retaining the scales of interest while removing scales that will be described statistically. In a canopy, these scales are different in horizontal and vertical directions and the appropriate region is a thin slab, as shown in Fig. 2.

In the horizontal direction, the resulting average field should be homogeneous over some distance while remaining representative of local conditions. For urban canopies it should be large enough to remove the dependency on exact positions of buildings and streets but small enough so as not to combine very different parts of a city. This scale is commonly referred to as the neighbourhood scale and has been discussed extensively in the literature on urban canopy flows (see Britter and Hanna 2003). In the vertical direction, all scales of variability are to be retained so the vertical extent of the region should be small compared to the scales over which the flow or the geometry varies. While the region can be arbitrarily thin, it is still beneficial to average over a volume rather than an area so as not to miss components of fluid–solid interfaces that are aligned with the averaging region. An averaging region with these dimensions results in averaged fields that can be considered horizontally homogeneous on a local scale and can therefore be described with one-dimensional profiles.

Potential issues occur, however, if the fluid–solid interface has surfaces that are perfectly horizontal. This could lead to discontinuities in averaged profiles, and the separation of scales in vertical direction is no longer guaranteed. While no realistic surface is perfectly horizontal, urban canopies are often approximated with idealized surfaces such as arrays of cubes so these issues deserve some attention. The issue of discontinuities in profiles or their derivatives has been addressed by Howes and Whitaker (1985). It can be avoided by introducing a slight curvature in the shape of the averaging region, i.e. defining it as an oblate spheroid rather than as a perfectly flat disc. Similarly, the separation of scales can be guaranteed by introducing a small tilt of the averaging region, with a slope that is much smaller than the scales of the flow but much larger than the thickness of the averaging region that can be made arbitrarily thin. The issues with perfectly flat surfaces are therefore not some fundamental problem with the volume averaging methodology, but rather represent a special case that can be regularized, as shown in Fig. 6. These fixes do, however, introduce a length scale over which the regularization occurs. In the limit of this length scale going to zero, some terms do have step changes or tend toward a Dirac delta function.

### Derivation of Double-Averaged Budget Profiles

Based on this averaging region and the averaging rules discussed in Sect. 2, double-averaged profiles can be derived from instantaneous budget equations. The basic procedure is to average each term in time and space and swap the order of averaging and differentiation, applying the spatial averaging theorem where terms have non-zero boundary conditions. Assuming steady state and horizontal homogeneity, the double-averages become a function of the vertical direction $z$ only and derivatives in $x$, $y$, and $t$ vanish. Finally, non-linear terms are expanded and terms with vanishing averages (such as the vertical velocity ${\left\langle {{\overline{ w }}} \right\rangle }_{_{ I}}$) are dropped.

As described in Sect. 2.7, non-linear terms can be split into four parts after double-averaging. For vertical advection terms in the form ${\left\langle {{\overline{ w \varphi }}} \right\rangle }_{_{ I}}$, the advection by the mean flow ${\left\langle {{\overline{ w }}} \right\rangle }_{_{ I}} {\left\langle {{\overline{ \varphi }}} \right\rangle }_{_{ I}}$ is zero since the mean vertical velocity is zero. The product ${{\overline{ {{\left\langle w \right\rangle }_{_{ I}}^\prime } {{\left\langle \varphi \right\rangle }_{_{ I}}^\prime } }}}$ can be understood as turbulent transport due to motions at scales larger than the averaging region and is expected to be small for spatial averaging at the neighbourhood scale. In the literature on canopy flows, this term is not separated from the turbulent transport due to small-scale motions ${\left\langle {{\overline{ {\widetilde{ w } ^\prime } {\widetilde{ \varphi } ^\prime } }}} \right\rangle }_{_{ I}}$, resulting in a single term ${\left\langle {{\overline{ {w^\prime } {\varphi ^\prime } }}} \right\rangle }_{_{ I}}$ for turbulent transport. The remaining term ${\left\langle \widetilde{ {{\overline{w}}} } \;\widetilde{ {{\overline{\varphi }}} } \right\rangle }_{_{ I}}$ representing spatial correlations in the mean flow is usually referred to as dispersive transport.

While it has been known for a long time that volume averaging introduces dispersive terms (Raupach and Shaw 1982), not much progress has been made on their interpretation or modelling. It is therefore not clear whether much is gained by separating them from the terms for turbulent transport since both are influenced by the impact of the surface geometry on flow dynamics. One advantage of separating the terms is that it provides a direct connection to the three-dimensional turbulent transport ${{\overline{ {w^\prime } {\varphi ^\prime } }}}$ that has been studied extensively in the context of Reynolds-averaged Navier–Stokes (RANS) modelling. Furthermore, separate transport terms can always be recombined to obtain spatio–temporal correlations.

### Momentum Balance

To derive a double-averaged budget equation for streamwise momentum, we start with the instantaneous streamwise momentum balance of the incompressible Navier–Stokes equations,

17 $$\begin{aligned} \frac{\partial \, u}{\partial t} + \frac{\partial \, (u\,u)}{\partial x} + \frac{\partial \, (u\,v)}{\partial y} + \frac{\partial \, (u\,w)}{\partial z} = f_x + \nu \, \nabla ^2 \, u - \frac{1}{\rho } \frac{\partial \, p}{\partial x} \, . \end{aligned}$$

Here, $x$, $y$, and $z$ are the streamwise, spanwise, and vertical directions while $u$, $v$, and $w$ are the respective velocity components. The density $\rho$ and the kinematic viscosity $\nu$ are assumed to be constant and we include a volumetric force term $f_x$. Since we assume horizontal homogeneity for mean variables, a mean pressure gradient has to be expressed as such a force in the following equations.

Performing the double-averaging as outlined in the previous section we derive the steady-state profiles of the momentum balance expressed in terms of superficial averages (similar to Eq. 2.5 in Yuan and Piomelli 2014),18As discussed earlier, we split the non-linear term into two components: one for the correlations in time and one for the correlations in space. Since the velocities are assumed zero at fluid–solid interfaces, this term does not produce a surface integral. The viscous term does produce an integral of the viscous drag at the fluid–solid interface in addition to the viscous transport along the mean velocity gradient. For urban canopy flows, both of these terms can usually be neglected. The mean transport is negligible for flows at high Reynolds numbers while the viscous drag is negligible for aerodynamically rough surfaces where pressure drag dominates. Body forces can be non-zero if a background pressure gradient is expressed as an equivalent body force or if gravity has a component in the $x$-direction, such as when the coordinate system is aligned with an inclined surface.

Similar forms of the momentum balance (18) are commonly found in the literature on plant canopy flows as well as in the urban literature. However, they are usually not quite equivalent. Where superficial averaging is used, the spatial decomposition is usually based on the superficial average as well. As discussed in Sect. 2.3, this definition is problematic. However, it happens to be the case that the averaged dispersive momentum flux ${\left\langle \widetilde{ {{\overline{u}}} } \,\widetilde{ {{\overline{w}}} } \right\rangle }_{_{ S}}$ is the same no matter whether the deviations are defined on the basis of the superficial or the intrinsic average. This is only true because we assume that ${\left\langle {{\overline{ w }}} \right\rangle }_{_{ S}} = {\left\langle {{\overline{ w }}} \right\rangle }_{_{ I}} =0$ everywhere and this is not the case for dispersive fluxes in general. The use of (18) with a spatial decomposition based on the superficial average becomes problematic if other non-linear terms are considered.

The momentum balance shown in (18) is also sometimes presented with a definition of the averaging region that corresponds to intrinsic rather than superficial averaging. For plant canopies this may be admissible since plants take up little space so the fluid volume $V_{f}$ is close to the total volume $V$ (Finnigan and Shaw 2008). Since this assumption is not usually made explicit for flow over vegetation (e.g. Finnigan 2000), (18) has sometimes erroneously been applied to intrinsic averages in urban canopies. The correct momentum balance for intrinsic averages can be derived from the relation ${\left\langle {{\overline{ \varphi }}} \right\rangle }_{_{ S}} = \varepsilon {\left\langle {{\overline{ \varphi }}} \right\rangle }_{_{ I}}$ (similar to Eq. 10 in Nikora et al. 2007),19and in this case, all three momentum fluxes—viscous, turbulent, and dispersive—produce additional contributions that can be understood as follows. If we consider two averaging regions at $z$ and $z+\delta z$, the difference between total integrated fluxes through the top and bottom regions contributes to the momentum balance between $z$ and $z+\delta z$. The intrinsic average of fluxes can be different not only because of changes in the total flux through each surface but also due to changes in the cross-sectional area occupied by solid elements. In other words, a decreasing flux either means that momentum is locally removed from the flow by some other process, or it means that the same momentum is now transported over a larger cross-section. In the latter case, the change in the intrinsically-averaged momentum flux is not balanced by a source or sink term. The new terms in the intrinsic momentum balance correct for this by counteracting gradients of intrinsic fluxes that are due to changes in the geometry and compensating for their contributions to the momentum budget.

### Integral Forms of the Momentum Balance

While force balances as presented in the previous section can be useful for formulating one-dimensional models, many applications related to boundary-layer turbulence focus on transport processes rather than forces. We obtain an equation for double-averaged momentum fluxes by integrating the momentum balance (18) in the vertical direction up to a height $H$ at which fluxes are assumed to vanish, some distance above the highest roughness element at $z=h_{max}$,20This equation for superficial momentum fluxes is relatively easy to interpret, since all terms correspond directly to terms in the superficial momentum balance. The left-hand side consists of the budget of all momentum sources and sinks above $z$, while the right-hand side consists of the downward fluxes of momentum at the current $z$-level. Since superficial averaging is used, both sides are normalized by an area that is constant with height.

For the intrinsic momentum balance, the situation is more complicated since there is more than one way of deriving an integral form. The first way is by dividing the superficial equation (20) by $\varepsilon$,21The interpretation of this expression for the contributions to the intrinsic momentum flux is similar to the interpretation of the superficial equation. Again the budget of momentum sources and sinks above $z$ is equal to the downward fluxes at $z$. This time, however, the terms are normalized by the cross-sectional fluid area at the current $z$-level rather than by a constant total area. The body forces gain additional $\varepsilon$-factors but the term is equivalent to integrating the forces over the total fluid region above $z$ and dividing by the fluid area at $z$. The viscous transport produces a geometric term with a similar role as in the intrinsic momentum balance above. The intrinsic velocity can change due to inclusion or exclusion of fluid volume rather than local velocity gradients. In this case, the geometric term corrects for such gradients of the mean velocity that do not affect the total viscous transport.

A drawback of this integral form of the momentum budget is that the derivatives of the terms do not correspond to the terms in the intrinsic momentum balance. In fact, the integral terms can vary even if there is no local contribution to the integral. Computing the derivative of each term results in a force balance without geometric corrections of the transport gradients and all other terms have to adjust accordingly. Therefore, we wish to consider a different equation for the intrinsic momentum flux obtained by integrating the intrinsic force balance (19) from $z$ to $H$,22In this integral version of the intrinsic momentum budget, the gradients do correspond to local contributions to the force balance. The magnitude of the source and sink terms, however, is difficult to interpret since the values are normalized by the variable fluid area before they are vertically integrated.

The integral form of the intrinsic momentum budget can therefore be defined such that either the magnitudes or the derivatives of the values are meaningful, but not both. Moreover, the overall shape of the profiles is difficult to interpret in both cases. In contrast, the profiles that make up the integral form of the superficial momentum budget have a shape that is straightforward to interpret, though values are less representative of the local conditions within the fluid. In fact, this appears to be a general pattern of the difference between the two types of averaging. Intrinsic averages are more directly related to local values while superficial averages produce profiles that are more meaningful globally.

### Scalar Transport

Similar to the momentum balance, a budget equation can be derived from the transport equation of a passive scalar $c$ with molecular diffusivity $k_c$ and source or sink term $S_c$,

23 $$\begin{aligned} \frac{\partial \, c}{\partial t} + \frac{\partial \, (c\,u)}{\partial x} + \frac{\partial \, (c\,v)}{\partial y} + \frac{\partial \, (c\,w)}{\partial z} = S_c + k_c \, \nabla ^2 \, c. \end{aligned}$$

The averaged equation is essentially the same as the one for streamwise momentum except that there is no equivalent of the pressure term. For superficial averaging, the budget is as follows,24As with the momentum equation, similar versions of this equation are commonly found in the literature on vegetation and urban canopies, but they are usually presented either with a definition of $\widetilde{ {{\overline{\varphi }}} }$ as departure from superficial averages or they are presented as valid for intrinsic averages. The double-averaged transport equation for intrinsically-averaged quantities has additional terms due to the variation of geometry (similar to Eq. 13 in Nikora et al. 2007),25

## Flow Over Cubes

**Fig. 3:**
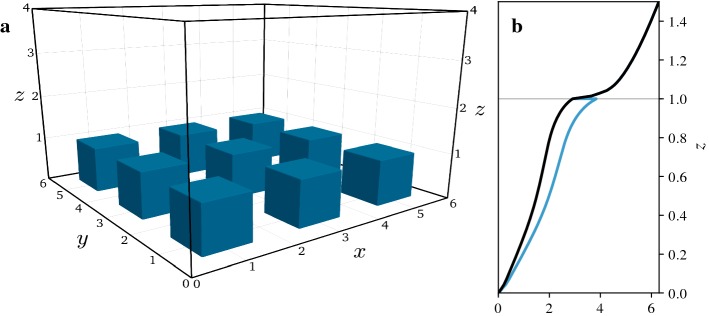
Large-eddy simulation of flow over constant-height cubes. Values are non-dimensionalized with the cube height $h$ and the friction velocity $u_\tau$. **a** Illustration of simulation domain. The $3\times 3$ cubes of size $h \times h \times h$ are arranged on a regular grid with periodic boundary conditions. The cubes are spaced at a distance of $h$, which results in a planar area fraction of $\lambda _p=0.25$ and a fluid volume fraction of $\varepsilon (z<h)=0.75$ within the canopy. **b** Resulting velocity profile of the superficial average ${\left\langle {{\overline{ u }}} \right\rangle }_{_{ S}}$ (black line) and the intrinsic average ${\left\langle {{\overline{ u }}} \right\rangle }_{_{ I}}$ (blue line) in the vicinity of the surface roughness

To further illustrate the behaviour and interpretation of spatially-averaged profiles, we consider a flow over cubes of constant height $h$ as shown in Fig. 3a. Surface-mounted cubes are commonly used as a simple idealization of real urban canopies, especially in the context of model development for the average profile. It is therefore useful to have a detailed understanding of the average profiles of such flows. In the following discussion we focus on the effects of non-negligible solid volume fractions on spatially-averaged profiles.

In the case of constant-height cubes, there is a single sharp transition in the volume fraction at $z=h$. As a result, averaged profiles of any quantity can be cleanly divided into a section without solid elements at $z>h$, a change in volume fraction at $z=h$, and a section with a reduced but constant volume fraction at $z<h$. This has the advantage that we can separate the effect of a transition in the fluid volume fraction on averaged profiles from more gradual changes in flow properties.

Above the canopy ($z>h$) superficial and intrinsic profiles are identical. As there are no contributions from surface integrals, the only complexity stemming from volume averaging is that spatial correlation terms such as dispersive momentum fluxes can be non-zero if mean fields are still influenced by the position of individual obstacles. At $z=h$, the fluid fraction $\varepsilon$ is reduced from $\varepsilon (z>h)=1$ to $\varepsilon (z<h)=1-\lambda _p$, where $\lambda _p$ is the planar area fraction occupied by cubes. While the sharp transition is useful to separate the effects of a change in fluid fraction from gradual changes within the flow, it has the downside that $\varepsilon (z)$ becomes discontinuous for a vanishing height of averaging region. For this reason, we first address the continuity of averaged profiles in Sect. 4.1 before we discuss the momentum balance across the transition in Sect. 4.2. Within the canopy ($z<h$) the fluid volume fraction is reduced but constant, resulting in intrinsic averages that are generally larger by a factor of $1/ \varepsilon$. The implications of this difference are discussed in Sect. 4.3.

We illustrate the discussion of the different terms with data from a large-eddy simulation (LES) of flow over aerodynamically rough cubes. The flow is driven by a constant pressure gradient and all values are non-dimensionalized with the cubes height $h$ and the friction velocity $u_\tau$. Details of the LES method employed can be found in Giometto et al. (2016), but we note that the following discussion is independent of numerical methods and quantitative results. The numerical results serve to illustrate the relationship between a turbulent three-dimensional flow field and the one-dimensional average profiles derived from it.

To simplify the comparison, transport terms are combined into a single stress tensor ${{\overline{\tau }}} ^{tot}$ that consists of a part that vanishes at the surfaces (turbulent and dispersive stresses in general, resolved turbulent and dispersive stresses in LES) and a part that does not (viscous stress in general, subgrid stress in LES).

For this set-up, the superficially-averaged momentum balance (18) and its intrinsically-averaged counterpart (19) simplify to the following expressions,

26 $$\begin{aligned}&- \frac{1}{\rho } \frac{\mathrm {d} \, {\left\langle {{\overline{ p }}} \right\rangle }_{_{ S}} }{\mathrm {d} x} - \frac{1}{\rho V } \int _{ A_{ f s} } {{\overline{p}}} \, n_x \, \mathrm {d}A - \frac{\mathrm {d} \, {\left\langle {{\overline{\tau }}} ^{tot} _{xz} \right\rangle }_{_{ S}}}{\mathrm {d} z} - \frac{1}{ V } \int _{ A_{ f s} } {{\overline{\tau }}} ^{tot} _{xn} \, \mathrm {d}A = 0 \end{aligned}$$

and

27 $$\begin{aligned}&- \frac{1}{\rho } \frac{\mathrm {d} \, {\left\langle {{\overline{ p }}} \right\rangle }_{_{ I}} }{\mathrm {d} x} - \frac{1}{\rho V_{f} } \int _{ A_{ f s} } {{\overline{p}}} \, n_x \, \mathrm {d}A - \frac{\mathrm {d} \, {\left\langle {{\overline{\tau }}} ^{tot} _{xz} \right\rangle }_{_{ I}}}{\mathrm {d} z} - \frac{{\left\langle {{\overline{\tau }}} ^{tot} _{xz} \right\rangle }_{_{ I}}}{ \varepsilon } \frac{\mathrm {d} \, \varepsilon }{\mathrm {d} z} - \frac{1}{ V_{f} } \int _{ A_{ f s} } {{\overline{\tau }}} ^{tot} _{xn} \, \mathrm {d}A = 0. \end{aligned}$$

The pressure here is a modified pressure that includes the subgrid kinetic energy.

**Fig. 4:**
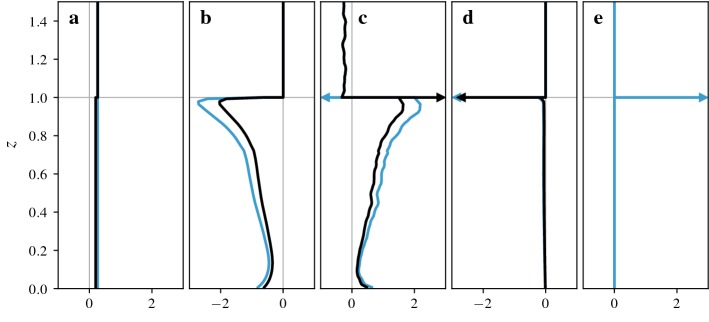
Superficial (black lines) and intrinsic (blue lines) momentum balance for flow over constant-height cubes. Arrows indicate singular contributions at $z=h$. **a** Constant pressure forcing $-1/\rho ~\mathrm {d}{\left\langle {{\overline{p}}} \right\rangle }_{_{ S}}/\mathrm {d}x$ and $-1/\rho ~\mathrm {d}{\left\langle {{\overline{p}}} \right\rangle }_{_{ I}}/\mathrm {d}x$. **b** Pressure drag $-1/(\rho V ) \int _{ A_{ f s} } {{\overline{p}}} n_x~\mathrm {d}A$ and $-1/(\rho V_{f} ) \int _{ A_{ f s} } {{\overline{p}}} n_x \mathrm {d}A$. **c** Gradient of momentum flux $-\mathrm {d}{\left\langle {{\overline{\tau }}} ^{tot} _{xz} \right\rangle }_{_{ S}}/\mathrm {d}z$ and $-\mathrm {d}{\left\langle {{\overline{\tau }}} ^{tot} _{xz} \right\rangle }_{_{ I}}/\mathrm {d}z$. **d** Skin friction $-1/ V \int _{ A_{ f s} } {{\overline{\tau }}} ^{tot} _{xn} \mathrm {d}A$ and $-1/ V_{f} \int _{ A_{ f s} } {{\overline{\tau }}} ^{tot} _{xn} \mathrm {d}A$. **e** Geometric correction term $-{\left\langle {{\overline{\tau }}} ^{tot} _{xz} \right\rangle }_{_{ I}}/ \varepsilon \;\mathrm {d} \varepsilon /\mathrm {d}z$ (only appears in intrinsic momentum balance)

**Fig. 5:**
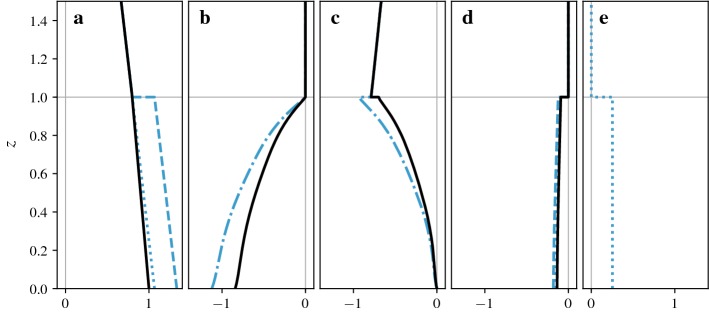
Integral form of superficial (black lines) and intrinsic (blue lines) momentum balance for flow over constant-height cubes. The dashed lines show the terms that are obtained by dividing the superficial balance by $\varepsilon (z)$ while the dotted lines are obtained by integrating the intrinsic momentum balance shown in Fig. 4. Dash-dotted lines show terms that are identical for both forms of the intrinsic balance. **a** Integrated pressure forcing $-1/\rho \int _z^H \mathrm {d}{\left\langle {{\overline{ p }}} \right\rangle }_{_{ S}} /\mathrm {d}x\;\mathrm {d}\hat{z}$ and $-1/(\rho \varepsilon ) \int _z^H \varepsilon \mathrm {d} {\left\langle {{\overline{ p }}} \right\rangle }_{_{ I}} /\mathrm {d}x\;\mathrm {d}\hat{z}$ or $-1/\rho \int _z^H \mathrm {d} {\left\langle {{\overline{ p }}} \right\rangle }_{_{ I}} /\mathrm {d}x\;\mathrm {d}\hat{z}$. **b** Integrated pressure drag $-1/(\rho V ) \int _z^{h_{max}} \int _{ A_{ f s} } {{\overline{p}}} \, n_x \mathrm {d}A \mathrm {d}\hat{z}$ and $-1/(\rho V_{f} ) \int _z^{h_{max}} \int _{ A_{ f s} } {{\overline{p}}} \, n_x \mathrm {d}A \mathrm {d}\hat{z}$ or $-\int _z^{h_{max}} 1/(\rho V_{f} ) \int _{ A_{ f s} } {{\overline{p}}} \, n_x \mathrm {d}A \mathrm {d}\hat{z}$ (intrinsic terms are equivalent for constant-height cubes). **c** Momentum flux ${\left\langle {{\overline{\tau }}} ^{tot} _{xz} \right\rangle }_{_{ S}}$ and ${\left\langle {{\overline{\tau }}} ^{tot} _{xz} \right\rangle }_{_{ I}}$. **d** Integrated skin friction $-1/ V \int _z^{h_{max}} \int _{ A_{ f s} } {{\overline{\tau }}} ^{tot} _{xn} \mathrm {d}A \mathrm {d}\hat{z}$ and $-1/ V_{f} \int _z^{h_{max}} \int _{ A_{ f s} } {{\overline{\tau }}} ^{tot} _{xn} \mathrm {d}A \mathrm {d}\hat{z}$ or $-\int _z^{h_{max}} 1/ V_{f} \int _{ A_{ f s} } {{\overline{\tau }}} ^{tot} _{xn} \mathrm {d}A \mathrm {d}\hat{z}$. **e** Integrated geometric correction term $-\int _z^{h_{max}} {\left\langle {{\overline{\tau }}} ^{tot} _{xz} \right\rangle }_{_{ I}}/ \varepsilon \;\mathrm {d} \varepsilon /\mathrm {d}\hat{z}\;\mathrm {d}\hat{z}$ (only appears in second version of intrinsic equation)

These force balances are shown in Fig. 4 while the resulting velocity profiles are shown in Fig. 3b. We also show the, perhaps more familiar, integral form of the momentum balance in Fig. 5, although one-dimensional models are usually based on the force balance and we do not have a unique definition of the integral equation for the intrinsic momentum flux.

As discussed in Sect. 3.4, there are two ways of defining the integral form of the intrinsic momentum balance. The first is obtained by dividing the integral form of the superficial balance by $\varepsilon$, the second is obtained by integrating the intrinsic force balance. While both contain the same intrinsic momentum flux term ${\left\langle {{\overline{\tau }}} ^{tot} _{xz} \right\rangle }_{_{ I}}$ (Fig. 5c), the other terms diverge at every change of volume fraction. When the volume fraction decreases, the integrals of the first balance are divided by a smaller cross-sectional area and increase. The second balance contains a separate term (Fig. 5e) with these contributions. Note that the only reason the profiles are identical for pressure drag (Fig. 5b) and almost identical for surface drag (Fig. 5d) is that these terms have no contributions above the step change in volume fraction at $z=h$. If there were multiple or gradual changes in volume fraction, the profiles would differ more significantly.

The overall shape of these LES profiles should be familiar from other simulations and measurements of canopy flows. Momentum added to the flow mainly above the canopy is transported down into the canopy by turbulent fluctuations where most of it is absorbed by pressure drag, as can be seen from the force balances (Fig. 4) and their integral forms (Fig. 5). The resulting velocity profile (Fig. 3b) has an inflection point at the level of the cubes and tends to a logarithmic shape above the canopy.

### Continuity and Differentiability of Profiles

If the averaging region is infinitesimally thin, the volume fraction has a discontinuity at $z=h$ where it undergoes a step change from $\varepsilon (h^+)=1$ to $\varepsilon (h^-)=1-\lambda _p$. Since the volume fraction relates the intrinsic and superficial averages, it follows that at least one of them has to be discontinuous too.

The continuity of averaged quantities depends on the boundary conditions at the cube tops. If a quantity is zero at the fluid–solid interface, the superficial average is continuous while the intrinsic average undergoes a step change, as is the case for the velocity profile in Fig. 3b. If the value of a quantity at the interface is statistically identical to values within the fluid, the intrinsic average is continuous instead. This is the case for a constant body force, as shown in Fig. 4a. Finally, there are quantities for which values at the boundary are non-zero, but statistically different from the values within the fluid, such as the vertical momentum fluxes shown in Fig. 5c. In this case, both the superficial and intrinsic averages are discontinuous.

Similar conditions apply for derivatives of profiles. Even though the superficial velocity in Fig. 3b is continuous, its derivatives are not, since the local velocity gradients are non-zero at the fluid–solid interface. It is therefore not obvious that terms such as $\mathrm {d}{\left\langle {{\overline{ u }}} \right\rangle }_{_{ S}} /\mathrm {d}z$ are meaningful and that we can write differential equations for ${\left\langle {{\overline{ u }}} \right\rangle }_{_{ S}}$.

As discussed in Sect. 3.1, the discontinuities are just an artefact of the idealized geometry and can be regularized with a small adjustment of the definition of the averaging region. We can, for example, consider an averaging region that is slightly inclined with one end being $\delta _z$ higher than the other end. This way, the profiles are continuous for any value of $\delta _z$, and as long as the height of the averaging region is much smaller than $\delta _z$, the separation of scales needed for non-linear terms is still provided. This $\delta _z$ can be made smaller than any relevant scale of the flow. In the limit of vanishing $\delta _z$, some terms can have a step change at $z=h$ or a singular contribution in the form of a Dirac delta function.

**Fig. 6:**
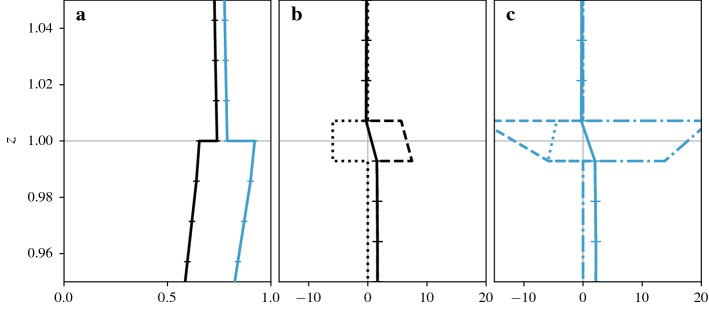
Details of transition at $z=h$. **a** Superficial momentum flux $-{\left\langle {{\overline{\tau }}} ^{tot} _{xz} \right\rangle }_{_{ S}}$ (black line) and intrinsic momentum flux $-{\left\langle {{\overline{\tau }}} ^{tot} _{xz} \right\rangle }_{_{ I}}$ (blue line). **b** Singular contributions to superficial momentum balance, regularized over a height equal to the grid size. The averaged stress gradient $-{\left\langle \mathrm {d} {{\overline{\tau }}} ^{tot} _{xz}/\mathrm {d}z \right\rangle }_{_{ S}}$ (solid line) is split into the gradient of the average stress $-\mathrm {d}{\left\langle {{\overline{\tau }}} ^{tot} _{xz} \right\rangle }_{_{ S}}/\mathrm {d}z$ (dashed line) and a surface integral $-1/ V \int _{ A_{ f s} } {{\overline{\tau }}} ^{tot} _{xz} \mathrm {d}A$ (dotted line). **c** Singular contributions to intrinsic momentum balance, regularized over a height equal to the grid size. The averaged stress gradient $-{\left\langle \mathrm {d} {{\overline{\tau }}} ^{tot} _{xz}/\mathrm {d}z \right\rangle }_{_{ I}}$ (solid line) is split into the gradient of the average stress $-\mathrm {d}{\left\langle {{\overline{\tau }}} ^{tot} _{xz} \right\rangle }_{_{ I}}/\mathrm {d}z$ (dashed line), a surface integral $-1/ V_{f} \int _{ A_{ f s} } {{\overline{\tau }}} ^{tot} _{xz} \mathrm {d}A$ (dotted line), and a geometric correction term $-{\left\langle {{\overline{\tau }}} ^{tot} _{xz} \right\rangle }_{_{ I}}/ \varepsilon \; \mathrm {d} \varepsilon /\mathrm {d}z$ (dash-dotted line)

It is important to note that the general behaviour of terms at $z=h$ is not a spurious effect of the idealized geometry. While profiles for more realistic surfaces would not become singular or discontinuous and would vary more gradually, they would show the same behaviour whenever the fluid volume changes. However, the effects of a gradually changing fluid volume fraction are more difficult to separate from gradual changes in flow properties within the fluid. The consequences of a reduction in fluid volume fraction are thus more readily demonstrated for the sharp transition of constant-height cubes, even if some profiles become discontinuous. For the singular contributions in Fig. 4 we also show regularized profiles in Fig. 6.

### Momentum Balance Across Transitions

At $z=h$, the profiles undergo several changes, some of which are related to the sudden addition of fluid–solid interfaces while others are related to the reduction in fluid volume fraction. The fluid–solid interfaces within the canopy are responsible for the onset of drag, as can be seen in Fig. 4b, d. This is linked to a change in sign of the gradient of momentum fluxes (see Fig. 4c), as now more momentum is removed by surface drag than is added by the pressure gradient. These effects are similar in urban and plant canopies as both canopies have a significant area of fluid–solid interfaces within the canopy.

Changes in fluid volume fraction, on the other hand, are a typical feature of urban canopies, but typically negligible in plant canopies. In general, it is a fundamental property of spatial averaging that it combines regions with different flow properties such as flow channelled into street canyons, wakes behind buildings, and boundary layers above horizontal surfaces. Averaged profiles obtain their shape not only from the changes within each region, but also from changes in the proportions of each flow regime within the averaging region. Whenever the fluid volume fraction decreases, flow regions just above horizontal surfaces are removed from consideration, resulting in systematic changes in averaged profiles.

In the case of constant-height cubes, this effect is quite evident. In regions just above cube tops, the flow feels the surface drag and is slowed to a halt. Above the gaps between the cubes, however, the flow is far from quiescent and there is a large downward momentum flux. When $z=h$ is reached, the average switches from representing both of those regimes to only representing the flow between cubes. This is true for both superficial and intrinsic averages, with the difference that the superficial average retains a contribution of zero for regions removed from consideration.

As a result, the velocity profiles in Fig. 3b show strong gradients just above $z=h$, as the gradients are dominated by the regions above the cube surfaces. Below $z=h$ the velocity gradients are reduced, although still steep. In effect, the average combines the more gradual changes of the flow entering the canopy with a sudden onset of a boundary layer at the cube tops. Similarly, there is a sudden change in the vertical flux of momentum, as shown in Fig. 5c. As fluxes are larger in regions above gaps between cubes compared to regions above cube surfaces, removing the cube tops from the average results in an increase of the intrinsic average while the superficial average decreases.

Since the momentum balance contains the vertical derivative of this sudden change in momentum flux, the contribution becomes singular as shown in Fig. 4c. For superficial averages, this term is balanced by the integral of surface stress over the top of the cubes (Fig. 4d); the momentum absorbed by the horizontal surface at $z=h$ results in a reduction of the momentum flux. For intrinsic averages, however, the momentum flux increases while the surface integral still produces a negative contribution. To close the momentum budget, an additional geometric term is required (Fig. 4e). The contributions to momentum fluxes across the transition are shown in more detail in Fig. 6.

### Scaling and Modelling Within Canopy

Within the canopy, where $z<h$, the volume fraction is reduced, but constant. Therefore, the terms of the intrinsic and superficial balance are identical up to a factor of $\varepsilon$. The shape of profiles is due to variations of flow quantities within the fluid region rather than variations in the geometry of the averaging region.

While the reduced volume fraction does not affect the shape of the profiles here, it still plays a role when comparing profiles from surfaces with different volume fractions. When comparing wind speeds within a densely packed canopy to wind speeds within a sparse canopy, for example, we can easily imagine a case where the intrinsically-averaged velocity is greater in the dense canopy while the superficially-averaged velocity is greater in the sparse canopy. Therefore it is possible to reach opposite conclusions based on the choice of averaging.

Similarly, the volume fraction is important when modelling averaged quantities in terms of different averages. Consider, for example, a typical model for the pressure drag,

28 $$\begin{aligned} D_p \equiv \frac{1}{\rho V } \int _{ A_{ f s} } {{\overline{p}}} \, n_x \, \mathrm {d}A \approx C_D \times {\left\langle {{\overline{u}}} \right\rangle }^2 \times \frac{1}{ V } \int _{ A_{ f s} } \frac{|n_x |}{2} \, \mathrm {d}A, \end{aligned}$$

where $C_D$ is a drag coefficient. Such a model could be based on the intrinsic average ${\left\langle {{\overline{ u }}} \right\rangle }_{_{ I}}$, the superficial average ${\left\langle {{\overline{ u }}} \right\rangle }_{_{ S}}$, or a combination thereof. It is an empirical question as to which of these choices results in a model that works best for surfaces with different packing densities.

One way to obtain a one-dimensional model with the correct scaling is by deriving it from a local, three-dimensional model. For example, it could be assumed that the turbulent momentum flux ${{\overline{ {u^\prime } {w^\prime } }}}$ is proportional to the local velocity gradient $\partial {{\overline{u}}} / \partial z$ with proportionality constant $\nu _\tau$. A first-order approximation for the spatial average (neglecting spatial correlations) would therefore be ${\left\langle {{\overline{ {u^\prime } {w^\prime } }}} \right\rangle }_{_{ I}} \approx {\left\langle \nu _\tau \right\rangle }_{_{ I}} {\left\langle \partial {{\overline{u}}} / \partial z \right\rangle }_{_{ I}}$ for intrinsic averages, but ${\left\langle {{\overline{ {u^\prime } {w^\prime } }}} \right\rangle }_{_{ S}} \approx \varepsilon ^{-1} {\left\langle \nu _\tau \right\rangle }_{_{ S}} {\left\langle \partial {{\overline{u}}} / \partial z \right\rangle }_{_{ S}}$ for superficial averages. The further assumption of a local proportionality between $\nu _\tau$ and $\partial {{\overline{u}}} /\partial z$ would result in ${\left\langle {{\overline{ {u^\prime } {w^\prime } }}} \right\rangle }_{_{ I}} \approx {\left\langle l_m \right\rangle }_{_{ I}}^2 {\left\langle \partial {{\overline{u}}} / \partial z \right\rangle }_{_{ I}}^2$ for intrinsic averages, but ${\left\langle {{\overline{ {u^\prime } {w^\prime } }}} \right\rangle }_{_{ S}} \approx \varepsilon ^{-3} {\left\langle l_m \right\rangle }_{_{ S}}^2 {\left\langle \partial {{\overline{u}}} / \partial z \right\rangle }_{_{ S}}^2$ for superficial averages, where $l_m$ is a local length scale. With the spatial averaging theorem, the mean velocity gradients can be related to gradients of mean velocity, resulting in

29 $$\begin{aligned} {\left\langle {{\overline{ {u^\prime } {w^\prime } }}} \right\rangle }_{_{ I}}&\approx {\left\langle l_m \right\rangle }_{_{ I}}^2 \left( \frac{\mathrm {d} \, {\left\langle u \right\rangle }_{_{ I}}}{\mathrm {d} z} + \frac{ {\left\langle {{\overline{ u }}} \right\rangle }_{_{ I}} }{ \varepsilon } \frac{\mathrm {d} \, \varepsilon }{\mathrm {d} z} \right) ^2 \end{aligned}$$

for intrinsic averages, and

30 $$\begin{aligned} {\left\langle {{\overline{ {u^\prime } {w^\prime } }}} \right\rangle }_{_{ S}}&\approx \varepsilon ^{-3} {\left\langle l_m \right\rangle }_{_{ S}}^2 \left( \frac{\mathrm {d} \, {\left\langle {{\overline{ u }}} \right\rangle }_{_{ S}} }{\mathrm {d} z} \right) ^2 \end{aligned}$$

for superficial averages. A model derived in such a way scales correctly between surfaces with different packing densities, relates the length scale of the global model ${\left\langle l_m \right\rangle }$ to a local length scale $l_m$, and allows for further improvements by including (and modelling) more of the neglected correlation terms.

## Discussion and Conclusion

When one-dimensional profiles of velocity and scalars in the urban canopy are considered, some form of averaging is usually implied. For statistically homogeneous flow fields, different forms of averaging become equivalent and implicit averaging can be understood as temporal, spatial, or ensemble averaging. Within canopies, however, profiles depend on spatial position even for long averaging times. Spatial averaging can remove this dependency on position, but it has to account for the solid parts of the averaging region.

The method of volume averaging has been investigated extensively for flow through porous media. In the context of porous media, many questions about details of the method have been resolved, such as the relation between averages of derivatives and derivatives of averages, the appropriate definition of spatial deviations, the differentiability of averaged quantities, and the assumptions made on a separation of spatial scales. By regarding one-dimensional profiles as a special case of this more general technique of volume averaging, these insights can be brought to the context of canopy flows.

Intrinsic and superficial averages, related through the fluid volume fraction, are two common ways of defining volume averaging. They can be considered equivalent for plant canopies if the vegetation occupies only a negligible fraction of the canopy layer. For urban canopies with non-negligible solid volume they are distinct and follow different mathematical rules. While intrinsic averages are more representative of local conditions, budget equations take on a simpler form when based on superficial averages. The reason for this is that intrinsic averages vary not only when a quantity changes within a given volume, but also when the same quantity is contained in a different fluid volume. The former change affects global budgets while the latter does not. Still, any equation can be expressed in terms of any average as long as the derivation and interpretation are consistent.

Based on the definitions and properties of the method of volume averaging, one-dimensional budget equations can be derived from their three-dimensional equivalents. With an averaging region defined as a thin slab, the equations retain any variability in vertical direction while horizontal variations are removed from consideration. While these equations are similar to equations for plant canopies, the non-negligible solid volume fraction introduces a few additional concerns.

One such concern is that intrinsic and superficial averages scale differently with the packing density of a surface. This becomes relevant when comparing profiles from different surfaces, since a comparison of superficial averages might lead to different conclusions than a comparison of intrinsic averages if the packing densities are very different. The scaling is also important when averaged terms are modelled as a function of other averaged quantities. Depending on which averages are used, such a model may or may not be valid for a range of packing densities.

Another concern is that the fluid volume fraction undergoes changes within the domain whenever there are fluid–solid interfaces that are not perfectly vertical. In this case, the shape of average profiles is not only affected by changes within the fluid but also by changes in what parts of the flow are included in the average. These changes can be concentrated at one height, as is the case for constant-height cubes, or they can be more gradual throughout the canopy. In the former case, it is easy to ignore these effects or misattribute them to numerical issues since they only appear at an isolated location in the profiles. In the latter case, it is easy to misattribute these effects to physical processes within the flow field such as an increase in turbulent motions.

Finally, the profile of the fluid volume fraction can be discontinuous, resulting in discontinuous average profiles. This issue only affects idealized surfaces and even then it can be avoided with a slight adjustment of the definition of the averaging region. Still, it is worth keeping in mind that average profiles of flow over idealized surfaces can have very sharp gradients, especially if such surfaces are used to create and test models for the one-dimensional velocity profile.

Overall, we argue that the impact of spatial averaging should be treated as a leading-order effect whenever profiles within urban canopies are considered. This entails providing complete definitions for spatial averaging operations and ensuring that derivations of mean equations, models for mean quantities, and interpretations of mean profiles are consistent with those definitions. When deriving averaged equations, spatial deviations should be defined on the basis of the intrinsic average and mathematical properties of the averaging operation should be applied carefully to account for the solid volume fraction, in particular when computing intrinsic averages of gradients and superficial averages of products. When introducing models for spatially-averaged terms, the type of averaging should be chosen either through careful deliberation or by deriving one-dimensional models from three-dimensional ones to obtain the correct scaling for variations of the fluid volume fraction within the canopy and between different surfaces. Finally, the impact of spatial averaging should be kept in mind when interpreting magnitudes and gradients of mean profiles, especially when these depend on the choice of average. Spatial averaging combines regions with very different flow properties, so averaged profiles are not only influenced by changes within each region but also by changes in the proportion each type of region contributes to the average. With this study we advocate for a rigorous consideration of spatial averaging in urban canopies and provide the necessary background for future research on urban flows.

## References

[CR1] Anderson TB, Jackson R (1967). Fluid mechanical description of fluidized beds. Equations of motion. Ind Eng Chem Fundam.

[CR2] Ayotte KW, Finnigan JJ, Raupach MR (1999). A second-order closure for neutrally stratified vegetative canopy flows. Boundary-Layer Meteorol.

[CR3] Belcher SE (2005). Mixing and transport in urban areas. Philos Trans R Soci A Math Phys Eng Sci.

[CR4] Bou-Zeid E, Overney J, Rogers BD, Parlange MB (2009). The effects of building representation and clustering in large-eddy simulations of flows in urban canopies. Boundary-Layer Meteorol.

[CR5] Britter RE, Hanna SR (2003). Flow and dispersion in urban areas. Annu Rev Fluid Mech.

[CR6] Castro IP (2017). Are urban-canopy velocity profiles exponential?. Boundary-Layer Meteorol.

[CR7] Castro IP, Xie ZT, Fuka V, Robins AG, Carpentieri M, Hayden P, Hertwig D, Coceal O (2017). Measurements and computations of flow in an urban street system. Boundary-Layer Meteorol.

[CR8] Cionco RM (1965). A mathematical model for air flow in a vegetative canopy. J Appl Meteorol.

[CR9] Coceal O, Belcher SE (2004). A canopy model of mean winds through urban areas. Q J R Meteorol Soc.

[CR10] Coceal O, Thomas TG, Castro IP, Belcher SE (2006). Mean flow and turbulence statistics over groups of urban-like cubical obstacles. Boundary-Layer Meteorol.

[CR11] Darcy H (1856). Les fontaines publiques de la ville de Dijon.

[CR12] Finnigan J. J. (1985). Turbulent Transport in Flexible Plant Canopies. The Forest-Atmosphere Interaction.

[CR13] Finnigan JJ (2000). Turbulence in plant canopies. Annu Rev Fluid Mech.

[CR14] Finnigan JJ, Shaw RH (2008). Double-averaging methodology and its application to turbulent flow in and above vegetation canopies. Acta Geophys.

[CR15] Giometto MG, Christen A, Meneveau C, Fang J, Krafczyk M, Parlange MB (2016). Spatial characteristics of roughness sublayer mean flow and turbulence over a realistic urban surface. Boundary-Layer Meteorol.

[CR16] Giometto MG, Christen A, Egli PE, Schmid MF, Tooke RT, Coops NC, Parlange MB (2017). Effects of trees on mean wind, turbulence and momentum exchange within and above a real urban environment. Adv Water Resour.

[CR17] Gray WG (1975). A derivation of the equations for multi-phase transport. Chem Eng Sci.

[CR18] Grimmond CSB, Oke TR (1999). Aerodynamic properties of urban areas derived from analysis of surface form. J Appl Meteorol.

[CR19] Howes FA, Whitaker S (1985). The spatial averaging theorem revisited. Chem Eng Sci.

[CR20] Kanda M, Inagaki A, Miyamoto T, Gryschka M, Raasch S (2013). A new aerodynamic parametrization for real urban surfaces. Boundary-Layer Meteorol.

[CR21] Kono T, Ashie Y, Tamura T (2009) Derivation of spatially averaged momentum equations of urban canopy model using the concept of the immersed boundary method. In: Proceedings of the seventh international conference on urban climate (ICUC-7), Yokohama, Japan, 29 June–3 July 2009

[CR22] Lien FS, Yee E, Wilson JD (2005). Numerical modelling of the turbulent flow developing within and over a 3-D building array, part II: a mathematical foundation for a distributed drag force approach. Boundary-Layer Meteorol.

[CR23] Lumley JL (1970). Stochastic tools in turbulence. Applied mathematics and mechanics.

[CR24] Macdonald RW (2000). Modelling the mean velocity profile in the urban canopy layer. Boundary-Layer Meteorol.

[CR25] Martilli A, Santiago JL (2007). CFD simulation of airflow over a regular array of cubes. part II: analysis of spatial average properties. Boundary-Layer Meteorol.

[CR26] Mignot E, Barthélemy E, Hurther D (2008). Turbulent kinetic energy budget in a gravel-bed channel flow. Acta Geophys.

[CR27] Mignot E, Barthelemy E, Hurther D (2009). Double-averaging analysis and local flow characterization of near-bed turbulence in gravel-bed channel flows. J Fluid Mech.

[CR28] Nikora V, McEwan I, McLean S, Coleman S, Pokrajac D, Walters R (2007). Double-averaging concept for rough-bed open-channel and overland flows: theoretical background. J Hydraul Eng.

[CR29] Oke TR, Mills G, Christen A, Voogt JA (2017). Urban climates.

[CR30] Parlange MB, Eichinger WE, Albertson JD (1995). Regional scale evaporation and the atmospheric boundary layer. Rev Geophys.

[CR31] Pedras MHJ, de Lemos MJS (2000). On the definition of turbulent kinetic energy for flow in porous media. Int Commun Heat Mass Transf.

[CR32] Raupach MR, Shaw RH (1982). Averaging procedures for flow within vegetation canopies. Boundary-Layer Meteorol.

[CR33] Reynolds O (1895). On the dynamical theory of incompressible viscous fluids and the determination of the criterion. Philos Trans R Soc A.

[CR34] Rotach MW (1993). Turbulence close to a rough urban surface part I: Reynolds stress. Boundary-Layer Meteorol.

[CR35] Rotach MW (1993). Turbulence close to a rough urban surface part II: variances and gradients. Boundary-Layer Meteorol.

[CR36] Slattery JC (1967). Flow of viscoelastic fluids through porous media. AIChE J.

[CR37] Tennekes H, Lumley JL (1972). A first course in turbulence.

[CR38] Tseng YH, Meneveau C, Parlange MB (2006). Modeling flow around bluff bodies and predicting urban dispersion using large eddy simulation. Environ Sci Technol.

[CR39] Whitaker S (1967). Diffusion and dispersion in porous media. AIChE J.

[CR40] Whitaker S (1973). The transport equations for multi-phase systems. Chem Eng Sci.

[CR41] Whitaker S (1999). The method of volume averaging.

[CR42] Wilson NR, Shaw RH (1977). A higher order closure model for canopy flow. J Appl Meteorol.

[CR43] Xie ZT, Fuka V (2018). A note on spatial averaging and shear stresses within urban canopies. Boundary-Layer Meteorol.

[CR44] Yang XIA, Sadique J, Mittal R, Meneveau C (2016). Exponential roughness layer and analytical model for turbulent boundary layer flow over rectangular-prism roughness elements. J Fluid Mech.

[CR45] Yuan J, Piomelli U (2014). Roughness effects on the Reynolds stress budgets in near-wall turbulence. J Fluid Mech.

